# Label-Free Determination of Hemodynamic Parameters in the Microcirculaton with Third Harmonic Generation Microscopy

**DOI:** 10.1371/journal.pone.0099615

**Published:** 2014-06-16

**Authors:** Steffen Dietzel, Joachim Pircher, A. Katharina Nekolla, Mazhar Gull, André W. Brändli, Ulrich Pohl, Markus Rehberg

**Affiliations:** 1 Walter-Brendel-Zentrum für Experimentelle Medizin, Ludwig-Maximilians-Universität München, München, Germany; 2 SyNergy, Munich Cluster for Systems Neurology, München, Germany; 3 Deutsches Zentrum für Herz-Kreislaufforschung e.V., München, Germany; Maastricht University, Netherlands

## Abstract

Determination of blood flow velocity and related hemodynamic parameters is an important aspect of physiological studies which in many settings requires fluorescent labeling. Here we show that Third Harmonic Generation (THG) microscopy is a suitable tool for label-free intravital investigations of the microcirculation in widely-used physiological model systems. THG microscopy is a non-fluorescent multi-photon scanning technique combining the advantages of label-free imaging with restriction of signal generation to a focal spot. Blood flow was visualized and its velocity was measured in adult mouse cremaster muscle vessels, non-invasively in mouse ear vessels and in *Xenopus* tadpoles. In arterioles, THG line scanning allowed determination of the flow pulse velocity curve and hence the heart rate. By relocating the scan line we obtained velocity profiles through vessel diameters, allowing shear rate calculations. The cell free layer containing the glycocalyx was also visualized. Comparison of the current microscopic resolution with theoretical, diffraction limited resolution let us conclude that an about sixty-fold THG signal intensity increase may be possible with future improved optics, optimized for 1200–1300 nm excitation. THG microscopy is compatible with simultaneous two-photon excited fluorescence detection. It thus also provides the opportunity to determine important hemodynamic parameters in parallel to common fluorescent observations without additional label.

## Introduction

Blood flow velocity is an important parameter in investigations of the hemodynamics of the microcirculation [1], [2] and various microscopic methods have been developed to measure it [2], [3]. The probably oldest account of intravital microscopy from 1634 already describes the movement of the heart and the flowing blood in a louse [4], [5], published nearly thirty years before Marcello Malpighi observed the circulation in blood capillaries in 1661 [5]. Bright field techniques permit label-free measurements in transparent tissues [6] or, with epi-illuminiaton, close to the surface [7]. Injection of fluorescent beads [8] or a limited amount of fluorescently labeled erythrocytes [9], [10] allow measurements of individual particle velocities whereas injection of fluorescent macromolecules such as dextranes labels the plasma. Velocity measurements can then be performed on the non-fluorescent footprint caused by the unlabeled blood cells [11], [12].

The speed of laser scanning microscopes was usually too slow to record whole images at a frequency that would allow tracking of individual flowing cells. In line scanning mode the laser scans a single line e.g. along the vessel center with high frequency. When a particle flows along this line, the progressing signal or footprint allows for velocity measurements. Assuming the observed vessel is parallel to the focal plane and has laminar flow, repeated measurements can give an accurate average blood flow velocity at the observed position. Axial line scanning for two-photon excited fluorescence was established using FITC-dextran labeling [11]–[13] and with fluorescent red blood cells (RBCs) [9]. In contrast to line scanning approaches, microscopic Particle Image Velocimetry (micro-PIV) is based on full field illumination. Velocity values for labeled particles are calculated from two images recorded with a short delay by cross-correlation. This procedure requires modeling that includes the application of several correction factors, e.g. for different magnifications and vessel diameters [14]. An approach that applied cross-correlation to scan lines from two-photon excited fluorescent dextran recently allowed blood velocity measurements of up to 84 mm/s in pathological arteriovenous shunts in the mouse brain [15].

The above examples show that line scanning is a well-established technique which was used so far with fluorescent labeling [9], [11]–[13], [15]. Labeling itself, however, has several disadvantages. Apart from the time and skill it requires to cannulate a suitable vessel, the injected volume may have a significant effect on cardiac preload and thus on the circulation in general. 100 µl with fluorescent dextran [16] represent about 5–10% of the total blood volume of a mouse. At early developmental stages, injection may be difficult or even impossible. Long-term observations may require repeated labeling since the label may extravasate (dextran) or get absorbed in the circulation (beads) with ill-defined physiological effects. In addition, plasma markers commonly used in animal experiments such as FITC- or TRITC-dextran are largely incompatible with GFP and other green or orange labels since their fluorescence is strongly overlapping. In any case, delivery of the labeled reagents to the vasculature by injection is an invasive procedure which should be avoided if possible. In particular with regard to a potential application in humans a label-free option for a detailed characterization of the microcirculation with flow velocity, shear stress and other parameters is highly desirable. In a first step towards this goal, we here explored Third Harmonic Generation microscopy in typical physiological models in mice and *Xenopus* as a label-free, potentially non-invasive alternative to determine hemodynamic parameters. Although non-fluorescent, it allows intravital microscopy with a signal intensity comparable to fluorescence. Since no label is needed, it offers minimal physiological interference with the examined tissue while enabling deep tissue penetration.

THG is an optical effect that is induced at the focal point of a pulsed laser by specific, inherent physical properties of a specimen [17], [18]. It thus combines the advantages of a label-free approach with restriction of signal generation to the focal spot. Just as two-photon excitation fluorescence microscopy, it permits high resolution 3D reconstruction of image volumes without out-of-focus background, several hundred micrometers deep into the tissue [19]–[22]. Fluorescence involves deposition of vibrational energy in the sample, the emitted photon has less energy than the excitation photon or the combined excitation photons in case of multi-photon excitation. In contrast, in THG an emitted photon carries the total energy of three incoming photons, with no deposition of energy in the sample. Since wavelength and energy are inversely correlated, the generated THG signal is at exactly one third of the wavelength of the irradiating laser. Therefore, a pulsed laser source with a wavelength of 1200 nm or higher is required to generate THG within the visible range (400–800 nm). THG signals with shorter wavelengths have been successfully recorded [23], [24] but since most optical glasses absorb UV light, such signals are attenuated if not all glass elements are UV transmissible.

Various conditions in the sample can lead to the generation of a THG signal. One possibility is a refraction index transition within the focal volume of the excitation laser, e.g. between a cell and its environment. Such refraction index transitions are also the source of contrast in classical microscopy techniques such as phase contrast and differential interference contrast, but these classical techniques cannot limit light emission to the focal plane only and are therefore not feasible in thick specimens. The absorption properties of hemoglobin provide another condition for strong THG signals and the first blurry THG microscopy images of isolated erythrocytes were published as early as 1999 [25]. This was later explained as resonantly enhanced THG caused by the absorption behavior of hemoglobin [26]–[28]. For example, erythrocytes produce a 425 nm THG signal when excited with 1275 nm pulsed laser light [22] because hemoglobin absorbs at 425 nm [28]. Images generated by THG microscopy are very different from those generated by the physically related Second Harmonic generation (SHG) [17], [19], [22], [29]. In SHG, a signal is obtained at half the wavelength of the irradiating pulsed laser source if a non-centrosymetric substance is present at a high density. This limits signal generation in mammalian soft tissues mostly to collagen fibers and striated muscle myosin. In previous work we visualized blood vessel walls and red blood cells in excised mouse cremaster muscle tissue by THG microscopy [22]. It remained unclear at the time whether THG microscopy is a valid option for dynamic intravital investigations such as blood flow velocity measurements.

With the current work we investigated if THG microscopy can be a feasible and reliable tool for a label-free intravital characterization of the microcirculation in established animal models. We found that such a characterization is indeed possible, e.g. by blood flow velocity measurements using THG imaging or THG line scanning and we describe several possibilities to evaluate such measurements. Controls using fluorescent beads validated THG blood flow velocity measurements. The cell free layer between flowing erythrocytes and the endothelium [30], [31] was visualized by fast recording of images as a gap between the THG signals of erythrocytes and the vessel wall. We further show that current optical equipment does not yet allow diffraction limited THG microscopy.

## Methods

### Specimen preparations

All animal experiments were performed according to German legislation for the protection of animals and approved by the Regierung von Oberbayern, München, Germany. For the mouse studies, protocols were approved under permits 55.2-1-54-2531-125-07 (cremaster preparations) and 55.2-1-54-2532-147-07 (ear investigations). For *Xenopus laevis* frogs, husbandry and breeding protocols were approved under permit 55.2-1-54-2531.6-3-10.

Black wild type C57BL/6 mice or albino-nude BALB/cAnNRj-nude mice were purchased from Charles River (Sulzfeld, Germany) or from Janvier (Saint Berthevin, France), respectively, and housed under conventional conditions with free access to food and water. The cremaster of male C57BL/6 mice at the age of 10–12 weeks was exposed as described [32] with minor modifications [22]. Mice were anesthetized using a ketamine/xylazine mixture (100 mg/kg ketamine and 10 mg/kg xylazine), administrated by intraperitoneal (i.p.) injection. For administration of FITC coupled 40 kDa Dextran (100 µl of 2.5 mg/ml; Sigma, Deisenhofen, Germany) the left femoral artery was cannulated in a retrograde manner.

Mouse ears were investigated after anesthesia induced by i.p. application of fentanyl (0.04 mg/kg; CuraMED Pharma GmbH, Karlsruhe, Germany), medetomidine (0.4 mg/kg; Pfizer GmbH, Berlin, Germany), and midazolam (4 mg/kg; Ratiopharm GmbH, Ulm, Germany). For microscopy, the procedure described in [33] was refined. The ear was gently fixed with a drop of vacuum grease (Baysilone-Paste, mittelviskos; GE Bayer Silicones, Momentive Performance Materials, Albany, New York) with the dorsal side of the ear up on a stack of microscopic glass slides held by a custom built-stage. The mouse body was placed on a heating pad to keep body temperature at 37°C. Suitable blood vessels were identified under transmission light from the halogen lamp and observation through the eye pieces. Arterioles and venules were identified on the basis of their blood flow direction and their general appearance. For bead measurements, yellow-green or polychromatic red fluorescent microspheres (diameter 1 µm, Polysciences, Eppelheim, Germany) diluted in 50 µL of PBS were injected via a tail vein catheter.

*In vitro* fertilizations, culture and anesthesia of wild type *Xenopus laevis* embryos was performed as previously described [34]. Developmental stages were determined according to [35]. Embryos were cultured in 0.1× MMR (0.1 M NaCl, 2 mM KCl, 1 mM MgSO_4_, 2 mM CaCl_2_, 5 mM HEPES, pH 7.8) at 22°C. Once embryos reached stage 20 (22 hrs post fertilization), the medium was replaced with 0.1× MMR supplemented with 0.2 mM PTU (*N*-phenylthiourea; Sigma-Aldrich, P7629) to suppress pigment formation in eyes and melanophores [36]. Stage 45 tadpoles (4 days post fertilization) were immobilized by anesthesia in 0.1× MMR containing 0.05% MS-222 (Ethyl 3-aminobenzoate methanesulfonate; Sigma-Aldrich, A5040) and THG microscopy was performed at room temperature.

For THG and two-photon excited fluorescence resolution measurements, respectively, titanium dioxide nanoparticles (anatase configuration, 25 nm; Sigma, #637254), dispersed with ultrasound in water as described [37] or fluorescent beads (deep red or orange, 0.17 µm, PS-Speck Microscope Point Source Kit, Molecular Probes – Life Technologies, Carlsbad, California, P-7220) were prepared in a 1% agarose gel cast in a chamber between a microscopic glass slide and a coverslip.

### Microscopy

The system used, a TriMScope (LaVision BioTec, Bielefeld, Germany), is described in detail elsewhere [22]. It was upgraded with Hamamatsu H7422A-40 high sensitivity GaAsP photomultipliers and equipped with a LaVision Imager 3 QE CCD camera for recordings of fluorescent beads. With 1275 nm excitation, the unattenuated intensity behind the XLUMPlanFl 20×/0.95W objective was measured with 190–220 mW.

Intravital microscopy was mostly performed with 90–100% of the unattenuated intensity. Black mice (C57BL/6), however have substantial amounts of melanin in their skin, partly evenly distributed and partly concentrated in spots. During intravital microscopy of the ear, the distributed fraction leads to attenuation of the excitation beam as well as of the generated signal while the spots strongly absorb the excitation light, leading to destruction of the surrounding tissue when irradiated with high laser powers. Thus, excitation intensity was reduced to usually 70–85% of the maximal intensity.

In some cremaster experiments, SHG and THG signals generated in forward direction were mirrored towards the backward detector by an aluminum coated coverslip below the tissue as described [29]. To maximize signal intensity, THG backward detection (447/60 filters) was close to the objective at the so-called ultra-sensitive port (USP) of the TriMScope, except when FITC-Dextran had to be recorded in parallel. Then the USP was used for FITC-detection (525/50) and THG was recorded at a standard port. A 624/40 Filter was used for SHG. THG in *Xenopus* tadpoles was recorded with forward detection.

### Image processing

Images were processed in ImageJ (http://imagej.nih.gov/ij/) or the derivate Fiji (http://fiji.sc/Fiji) and/or in Imaris 7 (Bitplane, Zürich, Switzerland). Point spread functions were calculated with the ImageJ macro “MIPs for PSFs all microscopes” kindly provided by Laurant Gelman, Basel (http://www.imaging-git.com/science/light-microscopy/routine-assessment-fluorescence-microscope-performance). Graphs and p-value calculations were made with Prism 6 (GraphPad Software, La Jolla, California). Figures for publication were assembled in Photoshop 9 (Adobe Systems, Mountain View, California). For presented micrographs, each color channel was mapped from the original 14 bit to 8 bit such that maximum detail was visible. No further processing was applied, except where explicitly noted in the respective figure legend.

### Blood flow velocity measurement in scanned images

**Figure 1:**
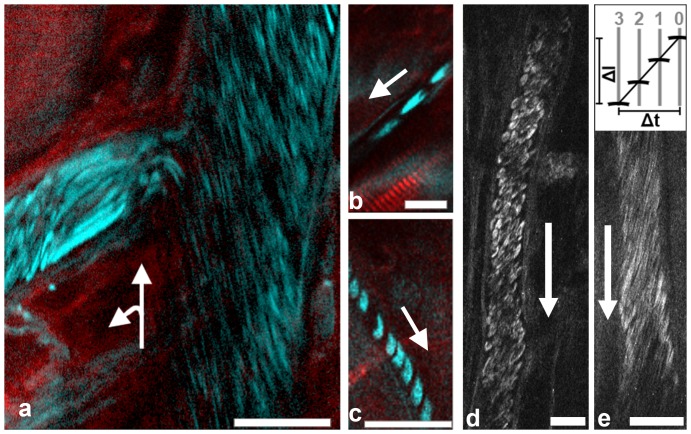
Intravital THG imaging of blood flow in the mouse cremaster muscle. Images were scanned with lines from top to bottom and line addition from right to left. Arrows indicate direction of blood flow. All scale bars 20 µm. (a) Combined SHG (red) and THG (cyan) image with mirror-enhanced signals [29]. In the left vessel, RBCs flow with the direction of added scan lines, therefore some are elongated to intense streaks. Image brightness was adjusted to allow simultaneous visualization of low and high intensities with a gamma value of 2. (b) Flow in this capillary was with the scan direction from right to left, therefore RBCs appear elongated. (c) Flow in this capillary was against scan direction, from left to right, RBCs therefore appear much shorter (compare scale bars). (d) RBCs flowing in a ∼25 µm vessel. RBC shapes in the image are optically deformed by the relation of blood flow velocity and the scanning process (see main text). (e) THG recording of RBCs suitable for blood flow velocity measurement. Scheme on top illustrates the principle with scanlines 0–3, see Methods for details. In the example shown scan speed was 800 lines per second, the temporal distance between two lines was thus 1.25 ms. Pixel size was 0.31 µm. Blood flow velocity was determined to be 0.75 mm/s.

Images produced with a point scanner such as a multi-photon microscope are assembled sequentially point by point and line by line, typically with a speed of several hundred lines per second. The width of an RBC spans several scanned lines. If it flows through the area of observation in parallel to the line direction, the speed of this object can be calculated from the displacement of the object's signal in the neighboring lines. In our microscope, each line is scanned from top to bottom, new lines are added to the left (scheme in Figure 1e). The micrograph in Figure 1e was scanned with 800 lines per second, the temporal distance between two lines thus being 1.25 ms. Pixel (px) size is 0.31 µm. A diagonal line created by the THG signal of a flowing RBC may cover 50 px height (15.5 µm) and 16 px width (20 ms), translating to a velocity of 0.78 mm/s. Since the approach requires that scan lines are parallel to the length of the vessel, either the scan head or the sample must be rotatable to investigate blood flow in vessels of arbitrary orientation.

### Manual blood flow velocity calculations on line scanning data

**Figure 2:**
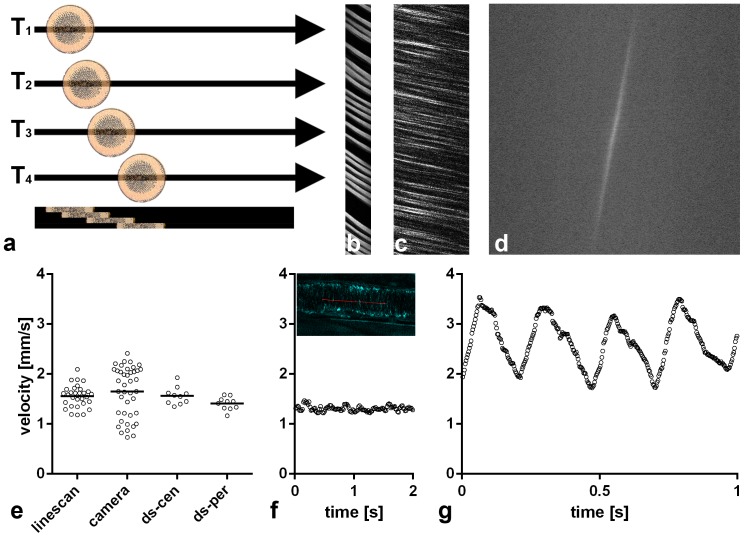
THG line scanning in the mouse cremaster muscle. (a) Scheme of the process. Erythrocytes passing by cause a signal in each of the four sequentially scanned lines, recorded at time points T_1_ to T_4_. Lines are put together in an x-t-representation (bottom). Here, each erythrocyte moving along the scanned line shows a continuously advancing position, resulting in a streak of signal with a measurable angle. The orientation of the individual lines is the same in all following x-t-representations. (b) x-t-representation from a capillary, (c–f): Comparison of various evaluations on the same 30 µm cremaster venule. (c) x-t-representation. (d) Fourier-transformation of the x-t-representation which is partly shown in c. The calculated average velocity in this example was 1.5 mm/s. (e) Comparison of velocities measured with THG line scan visualized in C (1.56 mm/s±0.22 s.d.) and with fluorescent beads and a camera (1.65 mm/s±0.50 s.d., Figure S1) in the same venule. Horizontal lines indicate mean values. Double line scans (ds) scanned two parallel lines alternately, with ds-cen more centrally (1.56 mm/s±0.17) and ds-per more peripherally (1.41 mm/s±0.12). Note higher velocity in ds-cen (p = 0.037, t-test). (f) LS-PIV measurements with the line scanning data set visualized in C. Two of seven seconds continuous run are shown with a data point every 16 ms. Average speed was 1.3 mm/s±0.07 s.d. The inset shows the position of the scan line (red) in this venule, visualized by THG. (g) LS-PIV measurements at the center of a 50 µm arteriole from the same cremaster muscle with a data point every 3 ms. Mean velocity over 11.9 seconds (19895 data points) was 2.35 mm/s, heart rate was ∼250 beats/min.

To calculate blood flow velocity from THG line scans, x-t representations (Figure 2b,c) were used. A streak caused by an RBC was selected. Assuming a rectangle with two opposing corners on this streak, the horizontal of the rectangle (Δx) indicates the covered distance and the vertical (Δt) the time needed. The angle α between the streak and the horizontal was determined by drawing a line selection on the streak in Fiji/ImageJ. The streak, Δx and Δt define a triangle. Δx and Δt are orthogonal to each other, therefore tan(α) = Δt/Δx and Δt = tan(α)*Δx. Velocity is v = Δx/Δt and therefore with the width W of a pixel along x and the loop time (from start of one scan line to the start of the next), the velocity is given as v = W/(tan(α)*[looptime]).

An x-t representation was subjected to a Fast Fourier Transformation in Fiji to calculate the average speed over many streaks, (Figure 2c). For semi-automated measurements, Fourier-transformed images were subjected to a Gaussian Filter (2 px width), a threshold was set and the thresholded region was selected via the wand tracing tool. ‘Fit ellipse’ was selected via the ‘set measurement’ command so that the ‘measure’ command would compute the angle β of the structure shown in the Fourier-transformed image, relative to the horizontal. For the formula given above, α = 90°−β. Autio et al. [38] used a related approach where they subjected a Fourier transformed image to an angle analysis to identify the mean RBC velocity.

### Blood flow velocity calculations on line scanning data with line by line correlation (LS-PIV)

RBC displacements between pairs of line-scans were determined using spatial cross-correlation analysis. They were computed with a MATLAB (The MathWorks, Inc) routine developed and generously made publicly available by Kim et al. [15]. The program was adapted in minor points such as to write results in a table for import in spreadsheet software, where the conversion from pixels to millimeters and seconds was performed. For arterioles, every 5^th^ time point was evaluated, for venules and capillaries every 10^th^. When the signal intensity decreased in the center of a vessel, the LS-PIV algorithm tended to calculate artificial velocities lower than in the vessel periphery. In some cases this could be avoided by careful visual inspection of x-t-diagrams and restriction of the evaluation to those areas of the scan line which showed visually detectable RBC streaks.

## Results

### Intravital THG imaging of flowing erythrocytes

The THG signal from RBCs in the microcirculation of the surgically prepared cremaster muscle was intense enough to allow intravital imaging with backward detectors, with or without signal enhancement by a mirror underneath the cremaster muscle [29] throughout the depth of this tissue, about 150–200 µm. However, in optical sections through larger vessels, the THG signal from RBCs was weaker towards the vessel center, most likely a consequence of absorption of the 425 nm signal by hemoglobin in RBCs above the focal plane. This effect limited detection of RBCs at the axis to vessels with a diameter of about 50 µm or less.

Flowing RBCs appeared elongated if moving with the direction of newly scanned lines since RBC velocity was fast compared to the scanning speed (Figure 1a, left vessel). Intravital recordings of capillaries allowed recognition of individual erythrocytes (Figure 1b,c) although they could not be followed with certainty from one time frame to the next.

We then positioned vessels parallel to the scan lines of the microscope to measure blood flow velocity by THG imaging. The center of the vessel in z-direction was easily determined by focusing on the vessel wall at maximal diameter. The RBC shape in the images depended on the relative speeds of blood flow and scanning. While a low flow/scanning-speed ratio resulted in deformed but recognizable RBCs (Figure 1d), only lines were detected in images with higher flow/scanning-speed ratios flow speeds (Figure 1e). The angle of these lines relative to the scanning direction and vessel axis allowed velocity measurements (Figure 1e, see Methods for details).

### Blood flow velocity measurement in vessels of the cremaster muscle with line scanning

As an alternative to imaging, a scanning microscope can repeatedly record a single line at frequencies over 1000 Hz. When a particle flows along this line, the progress of its signal allows for a velocity measurement (Figure 2a). Several studies applying line scanning with fluorescent markers were published and the line scanning applied here is consistent with these studies, except that to our knowledge we are the first to use a signal generated by a label-free technique. The scanned line was oriented parallel to the axis of the vessel and subsequent lines were visualized as x-t representations, showing the progression of THG-signal-generating RBCs along the line as a streak (Figure 2b,c). With the known scan frequency and the pixel size, the velocity of individual RBCs could be calculated from the angle found in such x-t representations for capillaries, (Figure 2b), venules (Figure 2c–f) and arteriole'1s (Figure 2g). As a second possibility to evaluate line scanning data, a Fourier transformation of the x-t-representation allowed fast calculation of the average velocity. (Figure 2d; see Methods for details).

In a third evaluation of THG line scanning data, we subjected line scan x-t representations to an automated spatial cross-correlation [15], a method named line-scan particle image velocimetry (LS-PIV) by the developers. Using the LS-PIV algorithm, we found good agreement with the values determined by evaluation of individual streaks from xt-representations (Figure 2e–f). In arterioles, this approach allowed to visualize the pulse velocity curve and thereby to calculate the heart rate (Figure 2g).

We then considered how to validate results obtained by THG line scanning with an independent approach. Line scanning with a fluorescent label such as FITC dextran would reproduce potential errors inherent to the line scanning approach. We therefore opted for fluorescent beads injected into the circulation and recorded them with normal epi-fluorescence microscopy and a CCD-Camera (Figure S1). Velocity was calculated from progression of beads in subsequent images recorded at about 20 Hz. In arterioles of the cremaster muscle, a large fraction of beads moved too fast (>3 mm/s) to allow measurements in camera images: the fluorescence intensity was smeared over a too long track and was thus so diluted that it could not be detected with sufficient certainty above background. Obtained bead velocities in venules were compared to THG line scanning results from the same vessel. Both techniques led to overlapping results, with a larger variability in bead velocity measurements compared to line scanning data (Figure 2e). Beads near the wall were slow, probably rolling along the wall. Beads flowing more centrally were faster and thus their fluorescence was spread over a longer track. Assuming laminar flow with velocities increasing from the periphery to the center, a THG line scan represents a specific fraction of the laminar layers. Therefore a single line scan can be expected to show less variation in velocity than beads from the whole cross-section of the vessel. The notion that velocity differences along the radius of the vessel caused the observed difference in variation between camera and line scans was supported by scanning two parallel lines at different radial positions of the vessel alternately. In such settings, the more central line showed higher velocities (Figure 2e).

### Line scanning measurements in vessels of the mouse ear

Any surgery performed on an intravital model may disturb the physiology of the observed tissue via inflammation or other trauma effects. For tests in a non-invasive model, we performed THG line scanning through the skin of the intact outer mouse ear (pinna). We imaged at the back side of the ear since we found vessels with a larger diameter (≥30) µm more frequently on this side of the central cartilage. The latter was scattering the laser beam too strong to allow excitation on its far side. All signals were recorded with backward detectors.

**Figure 3:**
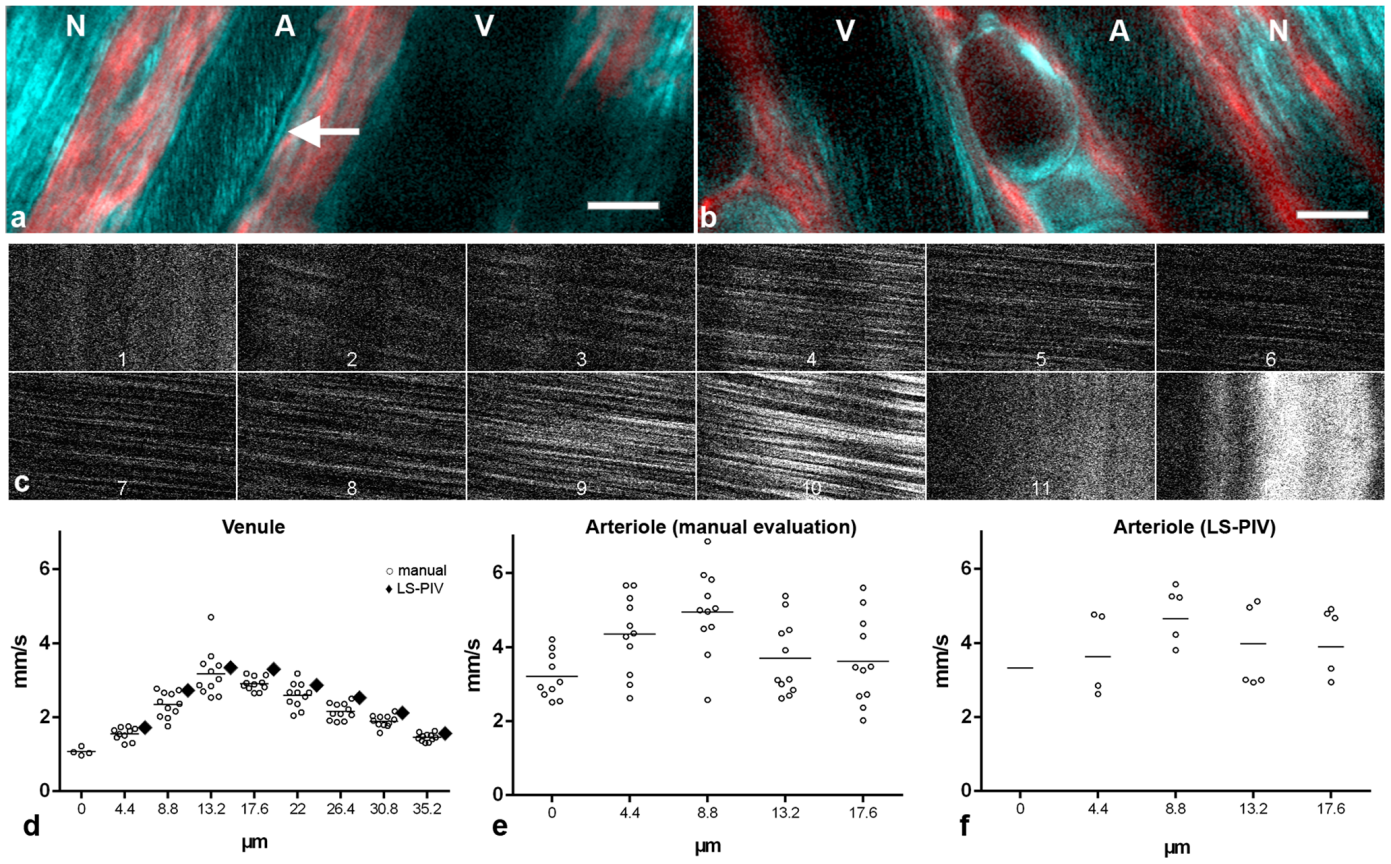
THG microscopy in the mouse ear. (a,b) Intravital imaging in different animals with arteriole (A), venule (V) and nerve fiber (N) running in parallel. In addition to the THG signal (cyan) SHG (red) is displayed. The arrow points to a typical linear vessel wall signal found only in arterioles. Scale bars 20 µm. The declining THG signal strength in more axial parts of a vessel are easiest to recognize in the arteriole in (a) and the venule in (b). (c–e) Flow velocity profile measurements by THG shifted line scan in the venule shown in b. (c) Clippings from x-t-representations of 12 parallel scan lines spaced 4.4 µm from each other. Lines 1, 11 and 12 were located in the vessel wall and thus show no movement. Note the shallower angles (faster velocity) and decreased intensity in the central scans. (d) Measurements derived from c by manual evaluation of individual streaks (circles) with mean values (horizontal lines) and mean values from LS-PIV calculations (diamonds). Scan lines 2 (0 µm) to 10 (35.2 µm) from (c) are included. Manually measured velocities revealed significant differences between the scan lines (p<0.0001, ANOVA) with significant differences between all direct neighbors (p<0.05 or smaller, post hoc Newman-Keuls test). For scan line 2 (0 µm), only a part of the x-t-representation showed blood flow. Apparently this line was close to the vessel wall so that slight movement could shift it outside. No reasonable LS-PIV average could thus be obtained. LS-PIV evaluations of the other scan lines confirmed significantly different blood flow velocities in general (p<0.0001; ANOVA, n = 88 for each line) and for all direct neighbors (****), except for the two central lines. (e) Measurements by manual evaluation for the arteriole shown in (b) Velocity measurements were significantly different between 0 and 4.4 µm lines (p = 0.0075) and between 8.8 and 13.2 µm lines (p = 0.0129). (f) LS-PIV results for the same arteriole. Only systolic maxima and diastolic minima (circles) and the mean values over all 3.2-millisecond-spaced 179 measurements (line) are shown. In the first scan line systole and diastole could not be identified reliably, therefore only the mean is given. Velocity differences between neighboring scan lines were all significant (p<0.0001) except between 13.2 and 17.6 µm (p = 0.87).

In ears of nude mice, intravital microscopy clearly showed THG signals from flowing red blood cells (Figure 3a,b). As in the cremaster muscle, the THG signal was weaker at the center of larger vessels (Figure 3a–c). This effect and not velocity itself limited measurements in larger vessels. By moving the scan line stepwise from one vessel wall to the other (Figure 3c), we obtained velocity profiles (Figure 3d–f) which showed 1.5× to 3× higher velocities in the center of the vessel compared to the periphery, as expected for a flow profile of laminar flow [2], [39], [40]. This approach again showed lower THG intensity in the vessel center (Figure 3c, panel 6 and 7). The exact position of the vessel lumen/wall border cannot be visualized simultaneously to axial line scanning. Assuming that in shifted line scans the border lines that show no movement (Figure 3c, panels 1 and 11) are located in the vessel wall, a minimal average shear rate at the wall can be calculated, e.g. 3.3 mm/s/4.5 µm = 733/s and 3.9 mm/s/4.5 µm = 866/s for the left and right sides of the arteriole in Figure 3f. The average shear rate between scan lines within this particular vessel varied between 17/s in the center and 229/s near the vessel wall.

Several-fold accelerations or decelerations of blood flow velocities were observed within seconds in some vessels, e.g. systolic maxima at the axial position decreasing in one arteriole from 6.1 to 1.7 mm/s within four seconds or raising from 4.0 to 9.4 mm/s within three seconds in another (Figure S2).

THG microscopy in the ears of black mice had to be performed at attenuated laser power, leading to diminished signals (see Methods). As in nude mice upon 1275 nm excitation, arterioles in the ears of black mice were outlined by an SHG signal at 638 nm from surrounding collagen. With 860 nm excitation, however, we did not obtain an SHG signal at 430 nm, demonstrating the strong absorption and/or scattering of blue light. Comparison of blood flow velocities measured either with THG line scanning or with fluorescent beads recorded by camera again led to overlapping results.

### Blood flow measurements in *Xenopus* tadpoles

**Figure 4:**
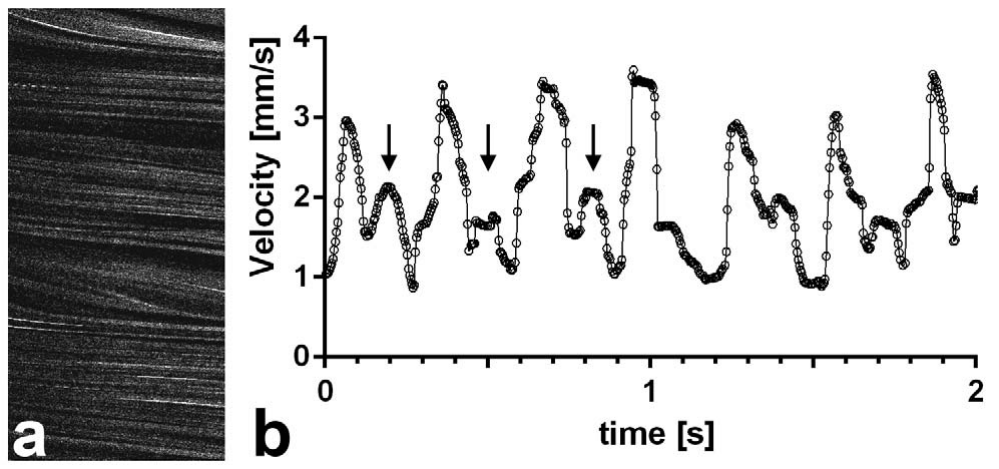
THG line scan measurement of the blood flow velocity in the tail artery of a stage 45 *Xenopus laevis* larvae (4 days post fertilization). (a) xt-representation showing 680 lines of the recording, representing the first 0.46 s of the graph in (b). (b) LS-PIV results from the two seconds depicted show values oscillating between 4 mm/s systolic and 0.9 mm/s diastolic. The higher peaks are determined by ventricular heart contractions. Arrows mark the first three secondary peaks (see main text). Calculation of the heart rate yielded a value of about 200 beats per minute, the vessel diameter was 45 µm.

To examine the feasibility of our THG line scan measurement procedure in another vertebrate model organism, we chose tadpoles of the African clawed frog *Xenopus laevis*. *Xenopus* wild type embryos were raised in the presence of *N*-phenylthiourea (PTU) to suppress pigment formation and anesthetized prior to microscopy (see Methods). Line-scan particle image velocimetry in tail vessels of stage 45 tadpoles revealed the presence of two sequential peaks (Figure 4). Hou and Burggren [41] described the sequential contraction of the ventricle and the conus arteriosus in *Xenopus*, causing two separate pressure peaks in arteries near the heart. An alternative cause for a second peak may be the reflection of the pulse wave in peripheral arteries [39]. The second peak we observed in the tail artery may be due to one of these phenomena or due to an overlay of both. A heart rate of about 200 beats per minute was calculated, which is similar to previous measurements of heart rates by visual inspection of the heart using conventional light microscopy (180+/−16 beats per minute, n = 10; R. Kälin and A.W. Brändli, unpublished observations).

### The cell free layer

**Figure 5:**
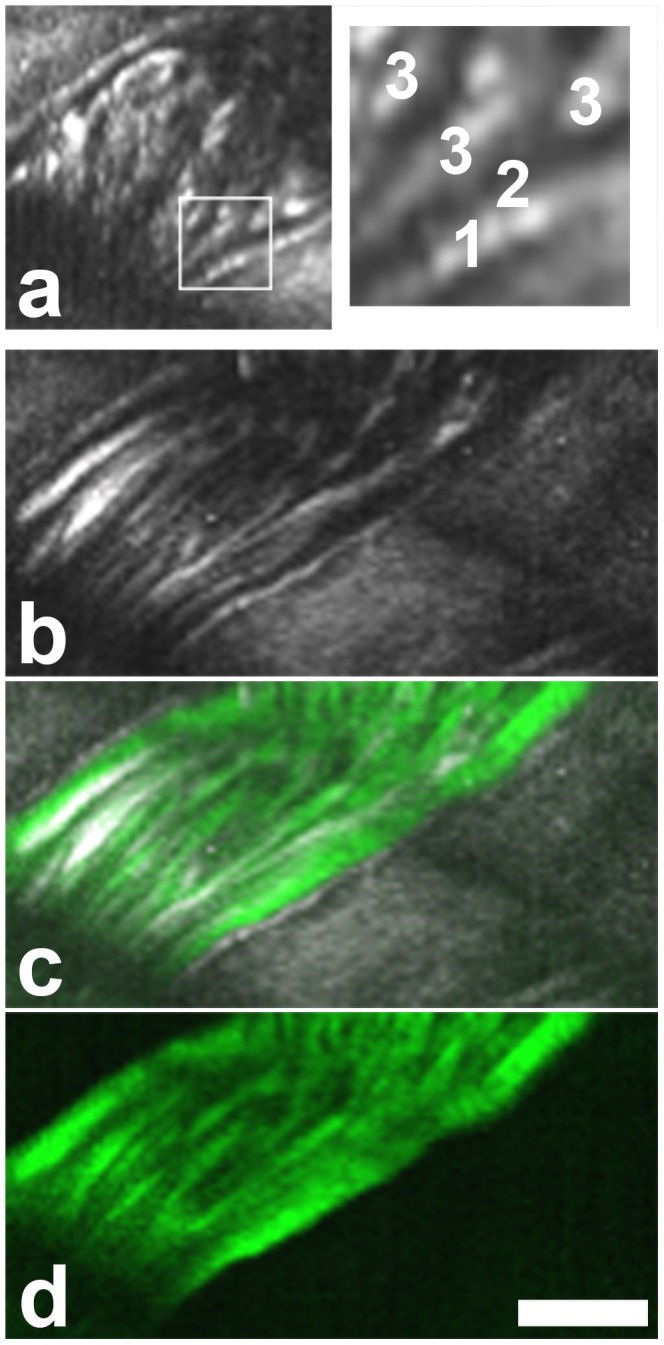
Visualization of the cell free layer in the cremaster muscle by THG with mirror-enhanced signals [29] **.** (a) RBCs are excluded from a gap near the vessel wall. Boxed area magnified on the right. 1, vessel wall. 2, THG free gap. 3, RBC tracks. (b–d) Image of the same area after injection of fluorescently labeled 40 kDa dextran. THG in gray (b,c), dextran fluorescence in green (c,d). The THG free gap is unchanged in width. Contrary to the erythrocytes, this small dextran infiltrates the cell free layer. Scale bar 20 µm. A movie of this vessel is shown in Movie S1.

A cell free layer was described in several intravital studies near the wall of blood vessels. This layer, also called endothelial surface layer (ESL), contains the glycocalyx, polymeric glycoproteins and associated plasma proteins produced by and attached to the endothelial cells [2], [30], [31], [42]. The cell free layer could be demonstrated in intravital THG microscopy of the mouse cremaster as a gap between the band of flowing RBCs and the THG signal of the vessel wall (Figure 5) During extended time series, occasionally an RBC track came in close contact with the vessel wall (Movie S1). It is not clear whether this was due to image distortions caused by animal movement or due to single RBCs entering the cell free layer. A vessel wall THG signal originating from the internal elastic lamina can be found in many arterioles (manuscript in preparation). The THG-free gap had an average width of 1.8 µm (s.e.m. ±0.2; average vessel diameter 32.1 µm ±5.7; n = 6). While RBCs are excluded from the endothelial surface layer, it was shown to be accessible for small molecules such as fluorescently labeled 40 kDa dextran [43]. When this compound was injected it stained the plasma in the vessel lumen, including the gap between vessel wall and the RBC tracks, showing that the THG-free gap is indeed part of the lumen. (Figure 5c, Movie S1). This argues against a significant contribution of the endothelial cells to the width of the THG-free gap at the given microscopic resolution.

### Theoretical and current actual spatial resolution of THG microscopy

While this study reveals new possibilities for microscopy of the microvasculature, we also encountered limitations such as insufficient signal brightness from the center of larger vessels (>50 µm). Current optical equipment is mostly not yet designed for use with wavelengths over 1200 nm as required for THG with a signal in the visible range. We therefore considered if current limitations could be overcome with optimized imaging equipment and compared present performance with theoretical limits. In multi-photon microscopy, spatial resolution depends on the size of the volume of the focal spot, which is measured as the full width half maximum (FWHM) of the point spread function (PSF) [44]. The theoretical, diffraction limited FWHM of a subresolution object in the focal plane (x,y) for a 3-photon excitation process is 0.51*λ/(√3 * NA) [45], [46] with λ being the excitation wavelength and NA the numerical aperture. For λ = 1275 nm and our NA = 0.95 objective we thus obtain a theoretical FWHM of 0.395 µm. In axial (z) direction, 0.88*λ/(√3 *(n−√(n^2^−NA^2^))) results in 1.622 µm (refractive index n_water_ = 1.33). However, the FWHM of the THG-PSF measured with test particles in agarose gel was 0.78 µm in x,y (standard deviation 0.08; n = 92) and 3.1 µm in z (s.d. 0.41) and thus in each direction about twice as wide as diffraction limited values would be. We found similar or worse deviations from diffraction limited values for other tested objectives.

The intensity of the THG signal depends on the density of excitation photons in the focal volume. All photons go through the waist of the illumination cone. Therefore the cross-section of this waist defines the photon density. Given by π*(FWHM/2)^2^, for 1275 nm a diffraction limited beam waist would have an area of 0.12 µm. However, it was 0.48 µm and thus four times larger. The likelihood that a three photon process such as THG occurs increases with the power of three of the photon density. This translates to a difference in signal brightness of 4^3^ = 64 times. We therefore should expect an about 60 times brighter signal with optimized, diffraction limited equipment, using the same amount of energy beamed through the sample. Theoretically, such a signal strength also could be achieved with current equipment and an excitation laser beam four times more powerful. However, apart from aggravated problems caused by increased absorption and photo damage, such a laser source is not available.

Measurements for several objectives showed a decrease in resolution when switching from wavelengths generated by the Ti:Sa laser to longer wavelengths generated by the optical parametric oscillator (OPO). To determine whether this was due to a sudden decrease in performance of optical elements at the longer wavelengths or due to beam profiles, we performed FWHM measurements for two-photon excitation fluorescence at 1025 nm with both beams. The FWHM of the PSF for TiSa was 0.46 µm in x,y (s.d. 0.08; n = 36) and 1.82 µm in z (s.d. 0.37) and for the OPO 0.99 µm in x,y (s.d. 0.08; n = 15) and 2.75 µm in z (s.d. 0.52). This shows that not only the objective but also the beam profile of the laser source, Ti:Sa or OPO, plays an important role in achieving diffraction limited resolution.

## Discussion

In this work we show that THG microscopy is a versatile tool for intravital determination of hemodynamic parameters of the microcirculation such as blood flow velocity, velocity pulse curve, velocity profiles, shear rate, vessel diameter as well as the heart rate. Proof-of-principle measurements were performed using widely used vertebrate animal models, the cremaster muscle and ear of adult mice as well as *Xenopus* tadpoles. Our findings demonstrate the suitability of the method for non-invasive label-free determination of hemodynamic parameters of blood vessels in both embryonic as well as adult tissues.

Label-free THG line scanning provides several advantages over previously established line scanning protocols using fluorescent labels [9], [11]–[13]. Foremost, it does not require any preparation except, if necessary, anesthesia. Introducing a catheter for application of a fluorescent label can be time consuming, not only requiring experimenter's skill and time but also prolonging anesthesia. In a non-invasive model such as the mouse ear [33], [47] or in chronic models such as the skin fold chamber [48], [49], this allows repeated THG-based measurements, limited only by anesthesia if required for the chosen model. In addition, potential physiological side effects of the label itself or its administration are avoided, as well as effects such as accumulation of FITC-dextran in macrophage-like cells over time.

Determination of hemodynamic parameters with THG microscopy can thus be performed as an alternative to methods requiring fluorescent labeling techniques, or it can be used in addition to fluorescent labels without blocking a color channel in the green or near read spectrum, which are typically used for fluorescence. This will allow a routine determination of hemodynamic parameters before, during and/or after intravital observations of structures labeled with fluorescent proteins [19] or other fluorophores, e.g. during observation of fluorescent immune cells [50]–[52] or blood platelets [53]. In addition, THG images allow observation of blood cells and vessels in their structural context. THG images (Figure 1a–c, Figure 3a,b) can be recorded alternating with line scanning (Figure 2a–c, Figure 3c) or fast imaging with a scan axis parallel to the blood flow can be applied (Figure 1d,e), where information about blood velocity and vessel structure are coded within the same image.

Line scanning, with THG or otherwise, would benefit greatly from respective tools in commercial acquisition software. Such tools should include easy application of shifted line scanning to determine velocity profiles over the vessel diameter (Figure 3c–f) and also an option for online calculation of an observed average blood flow velocity. A quick calculation could be performed from xt-diagrams (Figure 2b,c, Figure 3c) by a Fast Fourier Transformation (Figure 2d), since the necessary computing time is less than a second while a computation with the LS-PIV algorithm may take much longer. In addition, an estimation of blood volume flow could be included: for cylindrical vessels the volume flow can be calculated from measured vessel diameter and mean velocity. Mean velocity, in turn, can be estimated from center-line velocity [54].

To perform THG microscopy, all that is needed is a multi-photon microscope with an excitation wavelength three times larger than a detectable wavelength. Considering the low transmission of optics and the modest efficiency of detectors in the UV, this will usually mean an excitation source that produces wavelengths over 1200 nm. We here show that a current commercial setup allows THG line scanning for blood flow velocity measurements with an excitation of 1275 nm, although current equipment limits the applicability of THG microscopy in some environments due to insufficient signal intensity. In particular, the signal from the center of vessels with a diameter of 50 µm or more was too weak for reliable velocity measurements. The effect was independent of general depth within the tissue and was therefore specific for the optical properties within a blood vessel. While scattering by erythrocytes may be stronger than by muscle tissue, we assume that absorption of the signal by neighboring erythrocytes is the most relevant factor. This attenuation of the signal intensity but not high velocities limited THG velocity measurements in our current study. A signal intensity decrease towards the center of large vessels caused by hemoglobin absorption can be expected to be linearly dependent on vessel diameter. In contrast, improvements leading to a decrease of the THG excitation volume and thus a higher density of photons in that volume, will increase signal intensity with the third power, since THG is a three photon process. Therefore, already a modest decrease of this volume can be expected to substantially improve the signal-to-noise ratio. Our measurements and calculations show that we can expect an intensity increase of about 60 times once optical equipment is optimized and diffraction limited. In addition, optimized optics will have a high transparency for both, excitation and signal wavelengths, i.e. above 1200 nm and around 400–450 nm. Another option to reduce system aberrations is the use of adaptive optics which performs a pre-correction of optical aberrations by sending the excitation laser beam over a deformable mirror and thus keeps the excitation spot small and intense. This has been successfully used to maximize signal intensity for SHG and THG microscopy [55]. In addition, adaptive optics can pre-correct for optical aberrations generated by the observed tissue itself, aberrations which accumulate with increasing depth [55]. While we do not expect this approach to allow totally aberration free imaging, it should further decrease the actual excitation volume and thus improve signal intensity.

With regard to a potential application in human subjects, the question arises whether sufficient THG signal can be collected at excitation powers that are below the damage threshold. Examination of dermal layers [20] and the oral mucosa [56] with THG microscopy in human volunteers did not cause adverse side effects, suggesting that hemodynamic THG measurements will also be achievable in a clinical setting. Both studies visualized erythrocytes in capillaries, strengthening this notion. With optimized optical equipment, much deeper regions might be reached than with current technologies, which are limited to tissue areas near the surface. To this end, it may be advantageous to perform THG with longer wavelengths to minimize scattering in the tissue. As a light based technique, THG depends on a certain degree of optical transparency. It therefore will not be able to provide information on deeper layers of tissue that can be reached by some other methods that are used to investigate the microcirculation [57], such as ultrasound Doppler, without or with injected microbubbles as a contrast agent [58]. THG microscopy, however, does offer excellent spatial resolution at the subcellular level and morphological information on tissue context since line-scanning can be used alternating with imaging.

The literature is unequivocal concerning the existence of a cell free layer between the vessel wall and the flowing erythrocytes. There is quite some debate, however, on whether this layer is equivalent to the glycocalyx or mostly caused by axial migration of the blood cells. The latter is a physical effect which causes movement of RBCs to the central parts of a tube under flow conditions [59]–[61]. A number of studies have determined the thickness of the cell free layer in capillaries with an exclusion approach, measuring the distance between the vessel wall and RBC borders in bright field or the distance to a large fluorescent dye such as 70 kDa Dextran [30], [62], [63]. However, due to out-of-focus light and other optical difficulties related to fluorescence this approach was limited to vessels with a diameter of 15 µm or less [30]. We here show that with THG the exclusion approach can be transferred to larger vessels, since the THG signal originates only from the focal plane and both, erythrocytes and the walls of many arterioles cause sufficiently strong THG signals.

## Supporting Information

Figure S1 **Fluorescent beads travelling in a cremaster muscle venule.** Flow in the vessel is from right to left. Subsequent exposures have a time difference of 53 ms, composed of mostly exposure and a small readout time. Numbers indicate the start of the streak caused by the fluorescence of the bead and thus the position of the bead at the beginning of the exposure. At low signal to background ratios, comparison of the streaks in subsequent exposures may help to define the exact starting position of a streak. At the bottom right of each exposure is a bead that got stuck in a capillary in a slightly different focal plane. It can help to exclude moving artifacts. In later exposures another stuck bead can be seen at the center of the image. Scale bar 50 µm for all panels. (a) Two beads with average speed, 1.62 and 1.65 mm/s. (b) A bead with longer streaks and thus higher speed, 2.25 mm/s. (c) A slow bead, 0.76 mm/s, cyan numbers, can be tracked through many exposures. Towards the end a faster bead comes into view, with 1.86 mm/s.
(TIF)

Figure S2 **Changes of flow speeds in short time periods.** Two different arterioles in the same ear of a mouse were investigated. (a) Maximal systolic flow velocity decreases from 6.2 mm/s to 1.8 mm/s within four seconds. (b) Maximal systolic flow speed increases from 4 mm/s (at 3.2 seconds) to 9.4 mm/s (at 5.7 seconds) within 2.5 seconds.
(TIF)

Movie S1 **The cell free layer near the vessel wall.** The same sequence is shown twice, first with only the reflected THG signal and second the THG signal overlaid with the fluorescence from labeled dextran 40 kDa (green). Some frames are distorted due to movements of the animal. In a few frames an RBC track comes closer than usual to the vessel wall. It is not clear whether this is due to mentioned image distortions or due to single RBCs transcending the otherwise cell free layer.
(AVI)
