# Supplementary figures and images for: Label-Free Determination of Hemodynamic Parameters in the Microcirculaton with Third Harmonic Generation Microscopy
Source: PLoS One. 2014 Jun 16;9(6):e99615. doi: 10.1371/journal.pone.0099615 (PMC4059650; doi:10.1371/journal.pone.0099615)

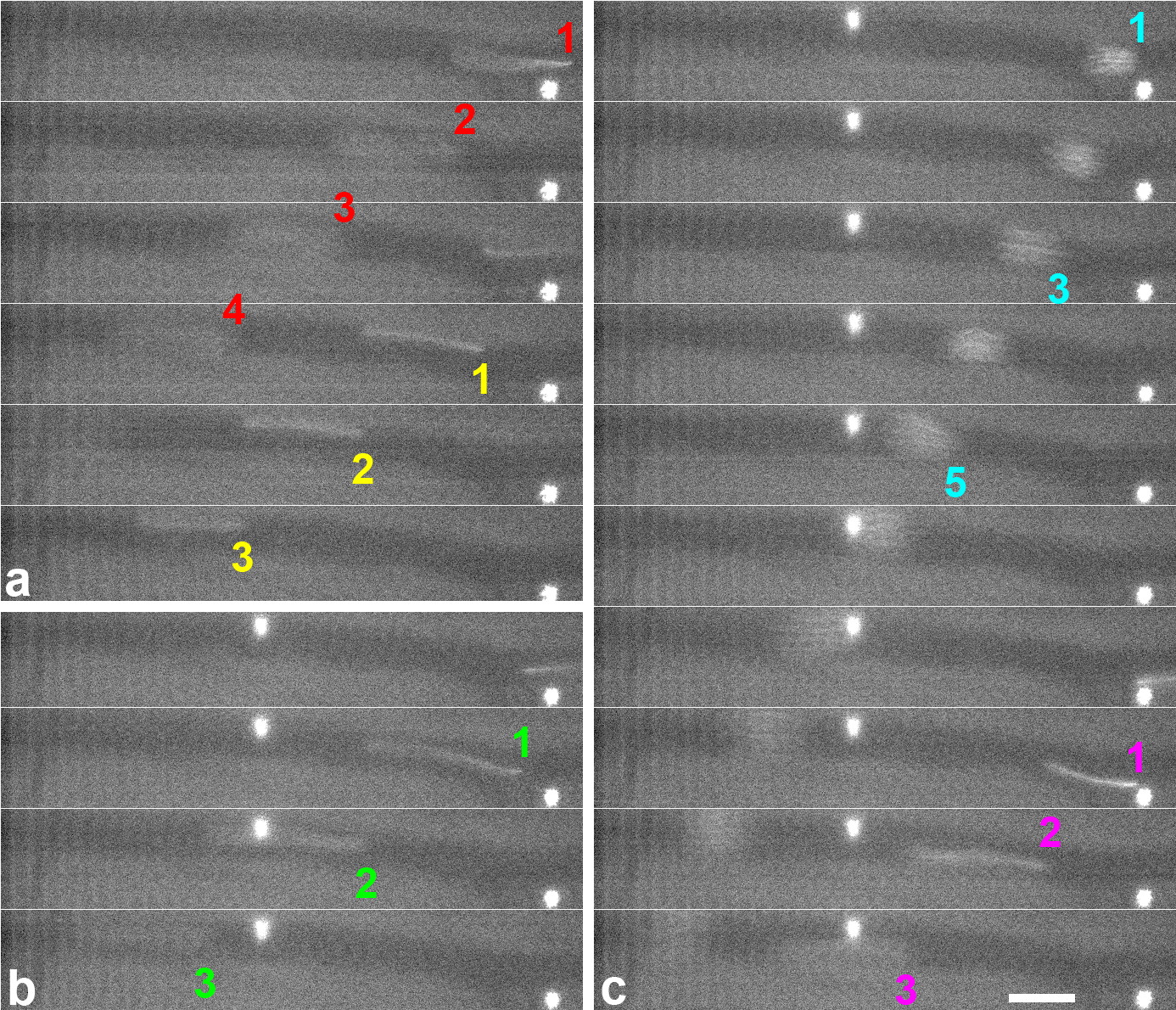

Supplement: Figure S1 — Fluorescent beads travelling in a cremaster muscle venule. Flow in the vessel is from right to left. Subsequent exposures have a time difference of 53 ms, composed of mostly exposure and a small readout time. Numbers indicate the start of the streak caused by the fluorescence of the bead and thus the position of the bead at the beginning of the exposure. At low signal to background ratios, comparison of the streaks in subsequent exposures may help to define the exact starting position of a streak. At the bottom right of each exposure is a bead that got stuck in a capillary in a slightly different focal plane. It can help to exclude moving artifacts. In later exposures another stuck bead can be seen at the center of the image. Scale bar 50 µm for all panels. (a) Two beads with average speed, 1.62 and 1.65 mm/s. (b) A bead with longer streaks and thus higher speed, 2.25 mm/s. (c) A slow bead, 0.76 mm/s, cyan numbers, can be tracked through many exposures. Towards the end a faster bead comes into view, with 1.86 mm/s. (TIF) [file pone.0099615.s001.tif]

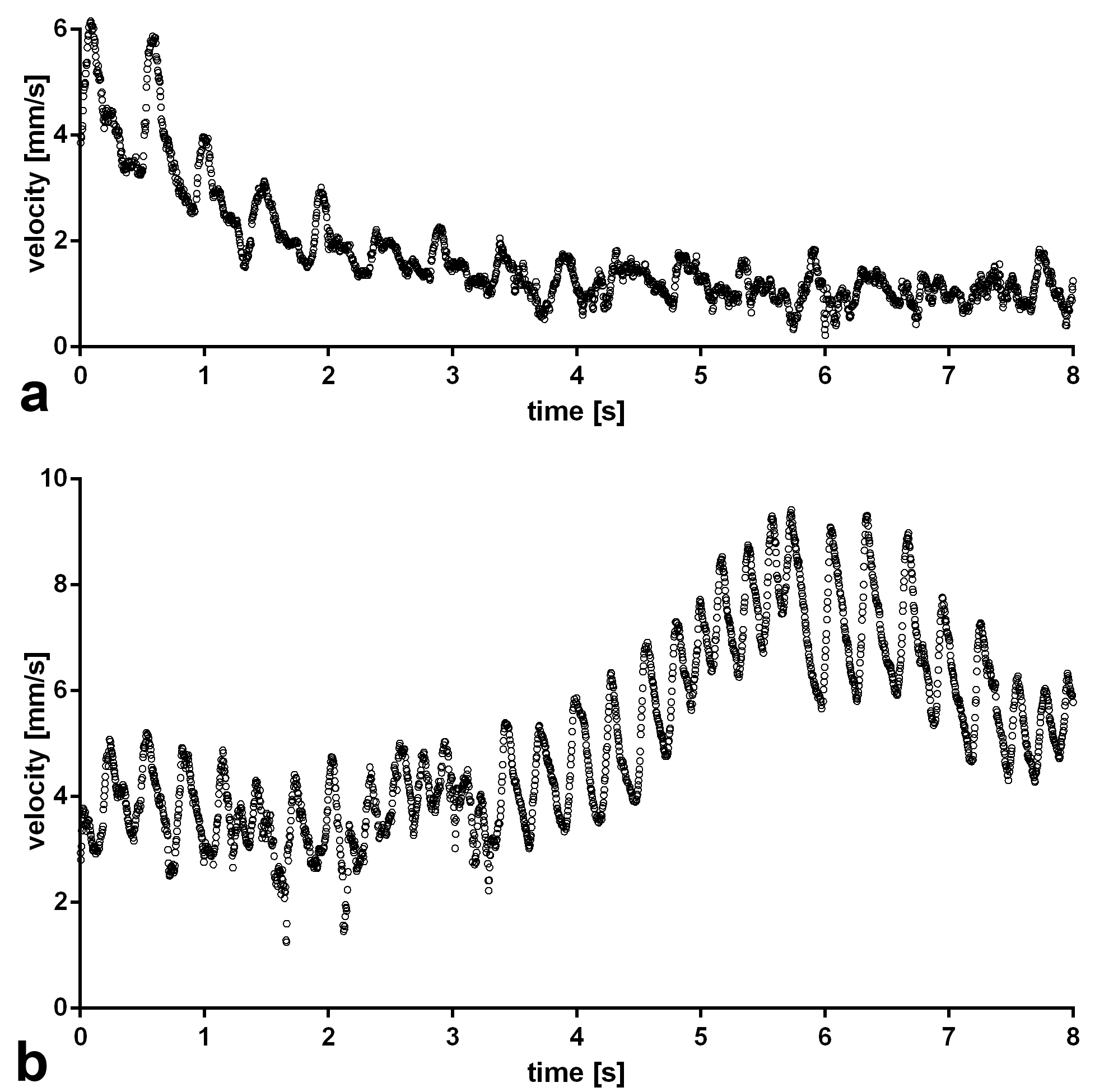

Supplement: Figure S2 — Changes of flow speeds in short time periods. Two different arterioles in the same ear of a mouse were investigated. (a) Maximal systolic flow velocity decreases from 6.2 mm/s to 1.8 mm/s within four seconds. (b) Maximal systolic flow speed increases from 4 mm/s (at 3.2 seconds) to 9.4 mm/s (at 5.7 seconds) within 2.5 seconds. (TIF) [file pone.0099615.s002.tif]
